# The Multifaceted Roles of Zinc in Neuronal Mitochondrial Dysfunction

**DOI:** 10.3390/biomedicines9050489

**Published:** 2021-04-29

**Authors:** Hilary Y. Liu, Jenna R. Gale, Ian J. Reynolds, John H. Weiss, Elias Aizenman

**Affiliations:** 1 Department of Neurobiology and Pittsburgh Institute for Neurodegenerative Diseases, University of Pittsburgh School of Medicine, Pittsburgh, PA 15261, USA; hyl36@pitt.edu (H.Y.L.); Gale.Jenna@medstudent.pitt.edu (J.R.G.); 2 YaghPenn Consulting, BV, 3061 Leefdaal, Belgium; ian.reynolds021@gmail.com; 3 Department of Neurology, School of Medicine, University of California, Irvine, CA 92697, USA; jweiss@uci.edu

**Keywords:** zinc, mitochondria, neurodegeneration, calcium, energy metabolism, mitochondrial dynamics

## Abstract

Zinc is a highly abundant cation in the brain, essential for cellular functions, including transcription, enzymatic activity, and cell signaling. However, zinc can also trigger injurious cascades in neurons, contributing to the pathology of neurodegenerative diseases. Mitochondria, critical for meeting the high energy demands of the central nervous system (CNS), are a principal target of the deleterious actions of zinc. An increasing body of work suggests that intracellular zinc can, under certain circumstances, contribute to neuronal damage by inhibiting mitochondrial energy processes, including dissipation of the mitochondrial membrane potential (MMP), leading to ATP depletion. Additional consequences of zinc-mediated mitochondrial damage include reactive oxygen species (ROS) generation, mitochondrial permeability transition, and excitotoxic calcium deregulation. Zinc can also induce mitochondrial fission, resulting in mitochondrial fragmentation, as well as inhibition of mitochondrial motility. Here, we review the known mechanisms responsible for the deleterious actions of zinc on the organelle, within the context of neuronal injury associated with neurodegenerative processes. Elucidating the critical contributions of zinc-induced mitochondrial defects to neurotoxicity and neurodegeneration may provide insight into novel therapeutic targets in the clinical setting.

## 1. Introduction

Zinc is a redox-inert divalent cation essential to a large number of biological processes, being the second most abundant trace element in the body, following iron [1]. Overall zinc levels are particularly high in the brain, where total zinc concentration may reach up to 150 µM [2]. Approximately 90% of the zinc present in the brain is protein-bound, contributing to the function of over 2000 proteins [3,4]. Indeed, zinc acts as a cofactor for over 300 enzymes, and, in the hippocampus alone, changes in cytosolic zinc can modulate the expression of over 900 genes [5,6], many of which are linked to the cell cycle, neurite extension, and synaptic growth [6,7,8,9]. Most of the remaining labile, nominally unbound or weakly bound zinc in the brain is present in synaptic vesicles of a large population of excitatory glutamatergic neurons throughout the cerebral cortex, hippocampus, striatum, and auditory brainstem [10,11,12]. Zinc is concentrated within synaptic vesicles by zinc transporter 3 (ZnT3) [13,14,15] and is synaptically released in an activity-dependent manner, acting as a neuromodulator for a number of neurotransmitter receptors [16,17,18,19,20]. Additionally, zinc released at glutamatergic synapses can translocate into postsynaptic cells through calcium-permeable α-amino-3-hydroxy-5-methyl-4-isoxazolepropionic acid receptors (CP-AMPARs) [21,22,23], voltage-gated calcium channels (VGCCs) [24,25], and N-methyl-D-aspartate receptors (NMDARs) [26], subsequently activating a number of physiological and pathophysiological signaling processes [19].

With the myriad cellular functions in which zinc plays an important role, disruption of zinc homeostasis can have severe detrimental effects. Nutritional zinc deficiency can lead to impaired brain development and cognitive dysfunction, including memory deficits and impaired learning ability later in life [27,28,29,30]. On the other hand, unregulated zinc release at glutamatergic synapses following traumatic brain injury (TBI), ischemia, and seizures has been implicated in excitotoxic damage and death of postsynaptic neurons [18,31,32,33,34]. Zinc dysregulation has been linked to increased risk for depression [35,36] and has been implicated in several neurodegenerative diseases, including Alzheimer’s disease (AD) [37,38,39,40,41,42], amyotrophic lateral sclerosis (ALS) [43,44], and Parkinson’s Disease (PD) [34,45,46,47]. Intracellular zinc concentrations are normally maintained at tightly controlled levels by a surprisingly large collection of fourteen SLC39a Zrt-/Irt-like protein transporters (ZIPs) and at least ten SLC30a zinc transporters (ZnTs), which transfer zinc into and out of the cytosol, respectively [1,48,49,50]. Intracellular zinc is also modulated by binding to cysteine-rich metallothioneins (MTs), which buffer and traffic the metal within cells [51,52]. Importantly, zinc liberated from MTs during oxidative stress conditions might represent a critical source for the metal in triggering several neurodegenerative changes [53,54,55,56]. Moreover, neuronal mitochondria may transiently contribute to the endogenous zinc pool [57], which can be released in response to injurious stimuli [57,58]. 

Zinc plays an important role in mitochondrial function, with neurons being notably reliant on this organelle to fulfill the high energy demands of the central nervous system (CNS). Indeed, it has been estimated that individual cortical neurons use approximately 4.7 billion ATP molecules per second compared to the 10 million ATP molecules used by non-neuronal cells in the human brain [59]. Under physiological conditions, zinc likely regulates mitochondrial processes in all tissues, including glycolysis, the tricarboxylic acid (TCA) cycle, and the electron transport chain (ETC) [60,61,62,63]. For instance, zinc increases the production of lactate and glycolytic intermediates in muscle cells and hepatocytes, indicating that it may act as an activating cation for phosphofructokinase (PFK) activity in glycolysis [62,63]. There is also evidence to suggest that hepatic zinc deficiency can inactivate mitochondrial biogenesis and DNA replication, leading to decreased expression of mitochondrial respiratory complexes I, III, and IV and decreased production of reactive oxygen species (ROS) [61]. In fact, dietary zinc protects against oxidative stress and ETC enzyme damage following postnatal protein malnutrition in rats [64]. Furthermore, in a mouse model of AD, dietary zinc supplementation has been shown to restore impaired mitochondrial respiration and increase levels of brain-derived neurotrophic factor (BDNF), which itself is linked to mitochondrial dynamics and oxidative efficiency [37,65,66,67]. The importance of zinc for energy metabolism is further supported by the fact that zinc chelation by N,N,N′,N′-tetrakis(2-pyridinylmethyl)-1,2-ethanediamine (TPEN) results in a 50% reduction of ATP production in hepatocytes [68].

However, zinc overload by entry through CP-AMPARs, VGCCs, and NMDARs at glutamatergic synapses, as well as release from intracellular zinc-binding proteins [40,53,63], may cause deleterious changes in intracellular zinc levels that contribute to neuronal injury and death, in part by inducing mitochondrial dysfunction [69,70,71,72,73,74,75]. Consequences of zinc-induced disruptions of cellular energy processes include decreased mitochondrial membrane potential (MMP), increased ROS generation, and mitochondrial permeability transition, resulting in the release of pro-apoptotic factors [71,74,76,77,78,79,80]. Synergism between zinc and calcium may exacerbate these effects [34,75,80,81,82,83,84,85]. There is also evidence to suggest that excess zinc may disrupt mitochondrial fusion, fission, and trafficking [86,87]. The overarching purpose of this review is thus to provide a comprehensive update of the current state of knowledge on the multifaceted effects of zinc on mitochondrial function and dynamics within the context of neuronal health, pointing to potential methods of protection against neurodegeneration. 

## 2. Zinc and Bioenergetic Function

Despite constituting only about 2% of human body weight, the brain utilizes approximately 25% of total glucose expenditure to meet its incredible energy demands [88]. The conversion of glucose and oxygen to ATP, carbon dioxide, and water takes place by glycolysis, the TCA cycle, and the ETC [88]. Glycolysis is a ten-step cytosolic pathway that converts glucose to pyruvate, ADP to ATP, and nicotinamide adenine dinucleotide (NAD^+^) to reduced nicotinamide adenine dinucleotide (NADH) [89,90,91]. Pyruvate is oxidized and utilized in the TCA cycle, a series of chemical reactions in the mitochondrial matrix that releases energy from acetyl-CoA [89,90,91]. This released energy is stored in electron carriers NADH and flavin adenine dinucleotide (FADH_2_), which are utilized by complexes of the ETC [89,90,91]. NADH donates its electrons to complex I, and FADH_2_ donates its electrons to complex II. These electrons are transferred in succession to ubiquinone (coenzyme Q), complex III, the mobile electron carrier cytochrome C, complex IV, and the final electron acceptor, oxygen [92]. Energy released from the ETC fuels the pumping of protons from the mitochondrial matrix to the intermembrane space (IMS), which establishes the proton motive force. The flow of protons down their concentration gradient drives ATP synthase, which catalyzes the phosphorylation of ADP to ATP [92]. Excess zinc has been shown to impair energy metabolism by inhibiting components of glycolysis [93], the TCA cycle [94,95], and the ETC [77].

### 2.1. Glycolysis

Elevated intracellular zinc has been shown to impair the glycolytic enzymes PFK and glyceraldehyde 3-phosphate dehydrogenase (GAPDH) [73,96,97]. PFK catalyzes the conversion of fructose 6-phosphate (F6P) and ATP to fructose 1,6-bisphosphate (FBP) and ADP [98]. GAPDH converts glyceraldehyde 3-phosphate (G3P) to 1,3-bisphosphoglycerate (1,3 BPG) and NAD^+^ to NADH [99]. There is evidence to suggest that zinc may directly inhibit GAPDH. Exposure of cortical neurons to neurotoxic levels of zinc results in a buildup of the upstream intermediates dihydroxyacetone phosphate (DHAP) and FBP, as well as a fall in ATP levels and subsequent cell death [93]. However, zinc inhibition of GAPDH may also occur by an indirect mechanism by which zinc induces a decrease in NAD^+^ levels, sufficient to inhibit GAPDH in and of itself. This decrease in NAD^+^ similarly leads to decreased ATP levels and neuronal death in cortical cultures exposed to zinc [93]. In support of this mechanism, the addition of pyruvate, which can regenerate NAD^+^ through lactic acid fermentation, rescues neurons exposed to toxic levels of zinc [93]. Notably, pyruvate provides protection against zinc toxicity in rat cortical cultures as well as reduced neuronal death and mortality in a rat model of ischemic injury [100]. 

Although there is no currently known pathology entirely attributed to zinc-mediated GAPDH inhibition, some neurological diseases have been associated with impaired GAPDH function. For instance, inhibition of GAPDH enzymatic activity causes dissociation of NAD^+^, which destabilizes GAPDH, making it prone to damage by denaturation and aggregation [101]. Denatured GAPDH binds to amyloidogenic proteins, which can contribute to the pathology of neurodegenerative diseases such as PD and AD [101,102,103]. Moreover, GAPDH aggregates in the brain have been proposed as a target for TBI treatment [104]. Of interest, functional GAPDH also promotes mitophagy of damaged mitochondria following cardiac ischemia. As such, disruption of GAPDH function may lead to the accumulation of damaged mitochondria and apoptosis [105], as observed in Huntington’s Disease (HD)-associated pathology [106]. 

### 2.2. TCA Cycle

Zinc impairment of the TCA cycle may occur by inhibition of the α-ketoglutarate dehydrogenase complex (KGDHC), a mitochondrial enzyme that catalyzes the conversion of α-ketoglutarate, NAD^+^, and CoA to succinyl-CoA, carbon dioxide, and NADH [73,94,107]. Zinc inhibition of the KGDHC may have significant pathological consequences, as deficiency of KGDHC activity has been linked to neurodegenerative diseases, including Friedreich’s ataxia, PD, and AD [108,109,110,111,112]. Zinc inhibition of the KGDHC likely occurs via the inactivation of lipoamide dehydrogenase (LADH) [73,78,113]. Indeed, zinc inhibits LADH with an IC_50_ near 0.15 µM in purified bovine enzyme, consistent with the predicted IC_50_ value for the KGDHC (0.1–0.4 µM) [94,113]. It is worth noting, however, that studies linking zinc to LADH inhibition were performed using isolated liver mitochondria and thus may not be necessarily translatable in neurons. Indeed, this is an important caveat to consider for all non-neuronal studies described in this review, as brain mitochondria have been shown to respond differently than organelles from other tissues, such as liver [114]. Nonetheless, it is of interest to investigate how zinc binds to and inhibits LADH in a neuronal preparation, and whether it contributes to persistently altered energy metabolism following neuronal injury [115,116]. 

Moreover, LADH is also a component of the pyruvate dehydrogenase complex (PDHC), which converts pyruvate, NAD^+^, and coenzyme A to acetyl-CoA, carbon dioxide, and NADH [107,117,118]. Interestingly, studies in SN56 cholinergic neuroblastoma cells suggest that zinc may inhibit the PDHC in a concentration-dependent manner, resulting in decreased acetyl-CoA levels and loss of cell viability [119,120,121,122]. Reduced activity of the PDHC has been associated with neuronal death following ischemia [118,123], leaving it open to speculation that zinc inhibition of LADH in the PDHC may play a role in neurodegenerative disease pathology. 

Mitochondrial aconitase (mACN), which catalyzes the conversion of citrate to isocitrate in the TCA cycle, is another potential mitochondrial site of zinc inhibition [95]. After toxic zinc exposure, albeit in hepatocytes, there was an observed buildup of citrate and decreased mitochondrial mACN expression, reflective of decreased mACN activity [95]. This is consistent with prior results indicating that zinc shifts equilibrium of the mACN-catalyzed reaction toward the reactant citrate in prostate secretory epithelial cells [124]. The exact mechanism of mACN inhibition is not yet fully elucidated, although it has been suggested to be the result of zinc displacement of iron at Fe-S sites of the enzyme [95]. Because zinc inhibition of other mitochondrial components causes MMP loss and ROS generation [77], we suggest that decreased mACN activity due to zinc may also be the result of these injurious processes. Indeed, mACN inactivation has been linked to MMP loss [125] and ROS accumulation [126]. However, it is worth noting that MMP loss was induced by excess calcium, not zinc, and the study was conducted using a human prostate cancer cell line, which, as noted earlier, may have significantly different biology than neurons [125,126].

### 2.3. Electron Transport Chain

Zinc has also been shown to inhibit the ETC, of which the mechanism and effects in mitochondria isolated from non-neuronal cell types [127,128] have been previously discussed [73]. Nonetheless, the effects of zinc on the ETC have also been demonstrated in rat brain cells [77]. Sub-micromolar levels of zinc have produced effects consistent with ETC inhibition, such as inhibited oxygen consumption and dissipated MMP [77]. The most probable site of zinc inhibition is complex III, as zinc binds with high affinity to a site in that complex that facilitates the carrying of protons across the inner mitochondrial membrane (IMM) [127,129]. Of relevance, complex III deficits have been linked to the development of numerous neurodegenerative disorders [130,131]. Moreover, complex III inhibition can increase glutamate release at the nerve terminal and contribute to HD-associated excitotoxicity [132,133,134]. 

Another putative target of zinc to consider is inhibition of complex I [135]. Even partial complex I inhibition can reduce nerve terminal oxygen consumption, increase glutamate release from synaptosomes, and potentially induce calcium-dependent excitotoxicity [136,137]. While zinc has been shown to inhibit complex I in bovine heart mitochondria, it is worth noting that this effect occurred at relatively high concentrations of the metal (10–50 µM), which may not be physiologically relevant [135]. However, one must consider the possibility that zinc inhibition of complex I could be of clinical significance at elevated microdomain concentrations during pathological conditions. It has been well established that complex I inhibition has been linked to neurodegenerative conditions, including brain ischemia/reperfusion injury [138], ALS [139], and PD [140,141,142]. 

## 3. Zinc and ROS Generation

One of the consequences of mitochondrial dysfunction is the generation of ROS, which are highly reactive molecules derived from oxygen [143,144]. Being byproducts of aerobic metabolic processes, most ROS products are formed by the reduction of oxygen to superoxide, which not only is heavily implicated in excitotoxic injury [145,146], but also serves as the precursor to other, perhaps more toxic ROS such as hydrogen peroxide, hydroxyl radicals, and peroxynitrite [143,147,148]. Although ROS can be generated by non-mitochondrial sources such as NADPH oxidase (NOX) [149,150], almost 90% of ROS are generated by the mitochondria [151,152,153,154,155]. Overproduction of ROS leads to oxidative stress, which in turn leads to the damage or destruction of proteins, lipids, genetic material, and cellular organelles [156]. Given the evidence for zinc-induced mitochondrial dysfunction and the fact that mitochondria are believed to be major generators of ROS in neurons, it is thus not surprising that zinc has been repeatedly shown to induce ROS production [70,71,77,157,158,159,160,161].

Zinc-induced mitochondrial ROS production has been demonstrated in mouse cortical cultures [71], possibly via inhibition of the LADH component of KGDHC in the TCA cycle [113,162]. LADH typically catalyzes the reduction of NAD^+^ to NADH. However, zinc inhibits LADH and accelerates the reverse reaction, favoring the oxidation of NADH by available oxygen in the mitochondrial matrix [113]. This reaction produces superoxide, which is converted to hydrogen peroxide by superoxide reductase. ETC components are also potential sources of mitochondrial ROS production. Zinc inhibition of complex III has also been posed as a likely cause of increased ROS levels in rat brain mitochondria [77]. This hypothesis is consistent with the finding that in mitochondria isolated from rat hearts, complex III inhibition seemed to be the main site for mitochondrial ROS production [163]. Inhibition of complex III using antimycin A resulted in increased ROS generation, an effect attenuated by preventing electron flow from complex I and II to complex III [163]. Together, these findings suggest that zinc-induced ROS production may be a result of zinc inhibition of TCA cycle and ETC components.

Non-mitochondrial sources of zinc-induced ROS generation have also been identified, such as the upregulation of NOX [159], a membrane protein that catalyzes the conversion of NADPH and oxygen to NADP^+^, a hydrogen ion, and superoxide [164]. A study in astrocytes concluded that zinc induces cell death primarily by increasing mitochondrial ROS production, but not by NOX activation, as inhibition of NOX-derived ROS reversed the neurotoxic effects of zinc to a lesser extent than inhibition of mitochondrial-derived ROS [70]. Non-mitochondrial ROS sources are nevertheless important due to the interplay between mitochondrial and non-mitochondrial ROS sources [158,160]. For example, zinc-induced NOX activation triggers mitochondrial ROS production in a HeLa cell model of hypoxia [160,161]. Conversely, zinc-induced mitochondrial ROS production causes NOX upregulation via activation of the NF-κB pathway in vascular smooth muscle cells [158]. Thus, mitochondrial and non-mitochondrial ROS generation may synergistically contribute to zinc-induced oxidative stress.

## 4. Zinc and Mitochondrial Permeability Transition

Another consequence of mitochondrial dysfunction is mitochondrial permeability transition, caused by the opening of the mitochondrial permeability transition pore (mPTP) [165], a relatively large, nonselective pore on the IMM [166]. mPTP opening occurs due to MMP loss, calcium deregulation, and oxidative stress [167]. Mitochondrial permeability transition causes the release of mitochondrial calcium stores, potentiating excitotoxic damage caused by elevated intracellular calcium [168,169,170] and resulting in the release of pro-apoptotic factors [171]. Mitochondrial permeability transition also allows for the mitochondrial influx of protons and other molecules smaller than 1.5 kD, leading to ETC uncoupling, MMP loss, and ATP depletion [172]. These effects may, in turn, induce ROS generation, amplifying mitochondrial permeability transition-induced cellular damage [167,173,174]. Importantly, excess zinc has been shown to be another trigger for mPTP opening [74,76,175].

Early evidence for zinc-induced mPTP opening showed that 2–10 µM zinc induces swelling of rat liver mitochondria, a marker of mitochondrial permeability transition [76]. In isolated brain mitochondria, zinc concentrations as low as 10 nM have been shown to cause effects consistent with mitochondrial permeability transition, including rapid mitochondrial swelling and release of the pro-apoptotic factors cytochrome C and apoptosis-inducing factor [74]. Although these studies suggest that zinc causes mitochondrial permeability transition by directly binding to and opening the mPTP, it is also possible that zinc may cause mitochondrial permeability transition indirectly by inhibiting enzymes of cellular energy processes and ROS production [78]. Indeed, zinc inhibition of matrix enzymes LADH, a component of KGDHC in the TCA cycle, and glutathione reductase (GR) and thioredoxin reductase (TR), which protect against oxidative stress, precedes changes associated with mPTP opening [78]. The extent and rate of zinc inhibition of these enzymes directly correlate with mPTP opening onset [78], suggesting that oxidative stress resulting from inhibition of these enzymes could induce mPTP opening [78,167].

Interestingly, a study comparing the effects of zinc and calcium on MMP loss in isolated liver mitochondria demonstrated that mPTP modulators reduced calcium-induced MMP loss but, surprisingly, did not attenuate the effects of zinc, suggesting that zinc does not open the mPTP [176]. Supporting this conclusion, the fluorophore calcein, a marker of mitochondrial permeability transition, was released from mitochondria treated with 30 µM calcium, but not with 30 µM zinc [176]. One potential explanation for the lack of mitochondrial permeability transition observed in this study [176] is the presence of zinc contamination in physiological solutions, such that low levels of zinc in control experiments may have already induced mitochondrial permeability transition, making additional zinc-mediated mitochondrial permeability transition difficult to observe [73]. A more likely explanation, however, is the absence of calcium in solutions utilized in experiments reporting a lack of zinc-induced mitochondrial permeability transition, since resting levels of calcium are required to initiate the opening of the mitochondrial calcium uniporter (MCU) [177,178]. The MCU is a likely conduit for zinc influx into the mitochondria, thereby initiating mitochondrial permeability transition [82], as discussed in more detail below.

## 5. Zinc Transport and Synergy with Calcium

Glutamate-induced calcium entry through NMDARs, CP-AMPARs, and VGCCs leads to changes in intracellular calcium that can result in excitotoxic neuronal cell death [34,179,180,181]. Mitochondria are a major target for this excitotoxic damage [182]. Calcium accumulation in the mitochondria, which occurs through the voltage-dependent anion-selective channel protein 1 (VDAC1) in the outer mitochondrial membrane (OMM) [183,184] and mitochondrial calcium uniporter in the IMM [185], causes impaired mitochondrial respiration, oxidative stress, mitochondrial permeability transition, and release of pro-apoptotic peptides [186]. Interestingly, due to the high affinity of calcium indicators for zinc [187], it has been suggested that some of the excitotoxic effects attributed to calcium may actually be caused by zinc [188]. Due to the development of higher-affinity zinc-selective indicators and low-affinity calcium indicators relatively unaffected by high zinc [189], the effects of these two ions on mitochondrial function can be investigated simultaneously. An increasing body of evidence using these improved detection methods suggests that excitotoxicity-induced mitochondrial dysfunction and subsequent cell death may be a result of the synergistic actions of zinc and calcium [34,75,80,81,82,83,84,85].

### 5.1. Synaptic Zinc

Zinc released at glutamatergic synapses enters the postsynaptic cell through CP-AMPARs [21,22,23], VGCCs [24,25], and, to some extent, NMDARs [26]. Interestingly, the type of channel through which synaptic zinc enters the cell may influence how the ion interacts with calcium and causes mitochondrial dysfunction. Whereas preferential zinc entry through CP-AMPARs causes acute ROS generation and MMP loss [71,79], smaller zinc loads caused by zinc entry through VGCCs cause more prolonged mitochondrial zinc accumulation and dysfunction [25,83]. The effects of this prolonged mitochondrial zinc accumulation may include mitochondrial swelling and release of pro-apoptotic factors [74]. The extended time durations associated with these mitochondrial defects are consistent with slow cell death after neuronal injury [190]. However, when associated with impaired cytosolic zinc buffering, as exhibited during ischemia, zinc entry through VGCCs can also cause acute mitochondrial dysfunction [85]. The presence of physiological levels of calcium caused greater mitochondrial dysfunction and neurotoxicity than zinc alone, suggesting calcium exacerbation of acute mitochondrial defects caused by zinc entry through VGCCs [85]. Following entry through CP-AMPARs, zinc has been shown to contribute to excitotoxic calcium deregulation and subsequent mitochondrial defects [31,80]. In support of zinc facilitation of calcium deregulation, small elevations in cytosolic zinc precede sharp calcium rises and MMP loss in CA1 neurons during oxygen-glucose deprivation (OGD), leading to subsequent cell death [80]. Furthermore, zinc chelation with TPEN significantly delayed the excitotoxic effects of calcium deregulation and facilitated the recovery of MMP [80]. 

The contribution of zinc to calcium deregulation likely takes place following zinc uptake into the mitochondria [80,191]. Numerous studies support the theory that mitochondrial zinc entry through the MCU is a precursor to zinc-induced mitochondrial dysfunction, calcium deregulation, and excitotoxic damage [75,79,80,82,84,192]. Indeed, blocking mitochondrial zinc entry through the MCU using ruthenium red (RR) has been shown to attenuate calcium deregulation [75]. Furthermore, neurons from MCU knockout mice exhibit significantly reduced mitochondrial zinc accumulation and protection against associated mitochondrial defects [82]. This attenuation of mitochondrial dysfunction and neurotoxicity was also demonstrated after wild-type neurons exposed to toxic levels of zinc were treated with RR [82]. In contrast to these data, a study found no evidence of mitochondrial zinc influx prior to mitochondrial depolarization [176]. However, as noted earlier, one potential explanation for the discrepancy is the lack of calcium in the experimental solutions used in this study, a requirement for activation of the MCU [177,178]. The fact that mitochondrial zinc entry is likely dependent on calcium underscores the synergistic relationship between zinc and calcium, where calcium-dependent mitochondrial zinc accumulation induces mitochondrial dysfunction and calcium deregulation [31,75,80]. Because zinc entry through the MCU contributes to calcium-induced excitotoxicity, inhibition of mitochondrial zinc accumulation may be an important potential target for neuroprotection after ischemia [82,192]. The MCU as a target for intervention after ischemia may be of particular promise in CA1 neurons, whose prolonged mitochondrial accumulation after reperfusion may contribute to their greater vulnerability than CA3 neurons to ischemic injury [193]. Of interest, the source of zinc damaging CA1 neurons following ischemia may be intracellular, rather than synaptic, in origin.

### 5.2. Cytosolic Zinc Pools

In addition to synaptic zinc entry through CP-AMPARs and VGCCs, zinc liberation from cytosolic pools such as MTs [53,54,55,56] can also cause rises in intracellular zinc that potentiate mitochondrial dysfunction and neuronal death [57]. In fact, after 2,2’-dithiodipyridine (DTDP)-induced zinc release from MTs, disrupting intracellular zinc buffering, far lower levels of zinc were required to cause mitochondrial dysfunction, ROS generation, and neuronal cell death [84,85]. As mentioned above, zinc liberation from metallothionein-III (MT-III) has been shown to be a major contributor to the increased susceptibility of CA1 neurons to ischemic injury compared to CA3 neurons [193], providing further support for the importance of intracellular zinc mobilization in neurodegeneration. 

Zinc release from MTs is typically thought to be caused by oxidative stress and acidosis [194,195]. Importantly, calcium entry through VGCCs or NMDARs and subsequent mitochondrial ROS generation may serve as an oxidative trigger for the mobilization of these cytosolic zinc pools [72]. Indeed, inhibition of calcium-induced mitochondrial depolarization and ROS formation by cyanidin-3-glucoside protected against glutamate-induced zinc release from intracellular pools in rat hippocampal neurons [196]. Interestingly, mitochondrial calcium entry may also be sufficient to cause zinc release from the mitochondria [83]. However, whether the mitochondria normally serve as an endogenous zinc pool without injury-mediated prior influx remains to be established.

### 5.3. Mitochondrial Zinc

There is significant colocalization of zinc and mitochondria following zinc loading [57]. Interestingly, in SN56 cholinergic neuroblastoma cells, about 12–20% of cellular zinc localized to the mitochondria, irrespective of zinc treatment conditions [191]. As previously discussed, mitochondrial zinc entry occurs through the MCU [75,79,80,82,84,192]. However, there may also exist MCU-independent mechanisms for mitochondrial zinc accumulation [197]. For instance, MTs have been observed to be localized in the IMS of isolated liver mitochondria [198], although it should be noted that MTs are absent from the IMS of isolated heart mitochondria [58]. However, MTs may localize to the matrix in mouse heart mitochondria, where they directly transfer zinc to mACN [199]. The localization of MTs in neurons has, unfortunately, not been adequately investigated.

An intriguing subject that has been poorly studied is the possible presence of zinc transporters (ZnTs and ZIPs) in neuronal mitochondria. Indeed, mitochondrial localization of ZnT2 has been demonstrated in mouse mammary cells [200], although a conflicting study found no colocalization of ZnT2 with mitochondria in human breast cancer cells [201]. Furthermore, ZnT1, ZnT4, and ZIP8 localize to mitochondria in rat hepatocytes [202], and ZIP7 and ZnT7 in rat cardiomyocytes [203]. Interestingly, a study using HeLa cells demonstrated that mitochondria-localized ZIP1 may associate with the MCU to regulate zinc influx into the mitochondria [204]. However, the distribution of ZnTs and ZIPs is highly tissue-dependent, and some of the mitochondrial zinc transporters investigated in these non-neuronal studies, such as ZnT2, may not be highly expressed in the brain [49,50]. Nonetheless, there are a number of ZnTs and ZIPs known to transport cellular zinc in the brain, such as ZnT1, ZnT3-ZnT6, ZIP1, and ZIP12 [1,48,49,50]. Thus, we believe that the potential mitochondrial localization of neuronal zinc transporters is an important component of neuronal mitochondrial zinc physiology that has yet to be adequately explored.

## 6. Zinc and Mitochondrial Dynamics

Mitochondria are highly dynamic, endosymbiotically-derived organelles [205] that undergo the opposing yet coordinated processes of fusion and fission [206,207]. Fusion and fission, which often occur quickly, simultaneously, and continuously, influence the morphology, number, and distribution of mitochondria in cells [208,209]. Fusion plays a role in repairing mitochondria during conditions of high energy demand [209,210,211,212,213,214], and fission mediates the selective destruction of damaged mitochondria by the process of mitophagy [215]. Fission also generates new, functional mitochondria [209] and may play a role in reducing oxidative stress-induced mitochondrial swelling and cell death in neurons [216]. Both fusion and fission are controlled by guanosine triphosphatases (GTPases), such as dynamin-related protein 1 (DRP1), mitofusin 1 (MFN1), mitofusin 2 (MFN2), and optic atrophy 1 (OPA1) [208,209,217]. Impaired fusion-fission balance caused by deregulation of these proteins can have detrimental effects on the mitochondrial function, morphology, and trafficking. Loss of fission impairs ETC complex assembly and oxidative phosphorylation [212,218], excess fusion results in mitochondrial elongation, and excess fission results in mitochondrial fragmentation [217,219]. Altered fusion and fission also disrupt mitochondrial transport to dendrites and axon terminals [220,221], where the highest neuronal activity occurs [222]. Driven by the high energy demands of growth cones and dendritic spines, mitochondria are trafficked to these distal areas [223,224,225], where they are required for axonal growth, development, and branching [66,226,227,228,229,230]. Impairments of mitochondrial trafficking cause energy depletion at typically mitochondria-dense sites, resulting in stunted dendritic development, decreased synaptic strength, reduced axonal regeneration, and neuronal cell death [229,231,232,233]. Both mitochondrial fragmentation caused by excess fission and impaired mitochondrial trafficking are implicated in the development of various neurodegenerative diseases [234,235,236,237]. 

### 6.1. Fission, Fusion, and Morphology

Zinc causes mitochondrial morphology changes such as mitochondrial fragmentation, evidenced by the formation of small, round mitochondrial puncta [238,239,240,241]. Zinc impairment of fusion–fission dynamics likely plays an important role in causing these morphological changes, as mitochondrial fragmentation and elongation are caused by excess fission and fusion, respectively [217,219]. Activation of DRP1, which plays a critical role in fission [242], has been shown to contribute to mitochondrial fragmentation, and recent studies have demonstrated that zinc causes mitochondrial fragmentation via DRP1 activation in ß cells [239,240,243]. Moreover, zinc induces DRP1-mediated fission, resulting in mitochondrial fragmentation and mitochondrial network disruption in brain endothelial cells [241]. Interestingly, several studies in ß cells, endothelial cells, and neurons suggest that DRP1 activation may be caused by ROS-induced transient receptor potential cation channel, subfamily M, member 2 (TRPM2)-mediated lysosomal zinc release, suggesting the existence of an ROS-zinc-DRP1 pathway that results in mitochondrial fission and fragmentation [240,241,243,244,245]. The mechanism for zinc recruitment of DRP1, however, remains unclear. Additionally, because several of these studies were conducted using non-neuronal cell types, it is of interest to investigate zinc-induced mitochondrial fission and fragmentation in neurons.

Independent from zinc recruitment of DRP1 to cause mitochondrial fragmentation, zinc may also play a role in DRP1-associated mitochondrial surveillance during the fission-mediated process of mitophagy in HeLa cells [204,246,247]. Following DRP1 recruitment, DRP1 interacts with the Zip1 component of an intriguing Zip1-MCU complex, allowing for mitochondrial zinc influx, leading to a reduction in MMP [204]. A novel model of mitochondrial surveillance suggests that this MMP loss is used as a stress test to segregate damaged mitochondria from healthy mitochondria [204]. According to this proposed form of mitochondrial surveillance, mitochondria that can re-establish their MMP are deemed healthy, whereas mitochondria that do not recover are eliminated by mitophagy [204]. The mechanism by which DRP1 interacts with Zip1-MCU to allow mitochondrial zinc influx is unclear. Furthermore, whether excess zinc interferes with this mitochondrial surveillance mechanism has not yet been demonstrated in neurons, although it is an intriguing topic of future study due to the extent that DRP1 activation and mitophagy have been implicated in the pathology of various neurodegenerative diseases [235], such as AD [234,248], PD [249,250,251], HD [252,253], ALS [254], and ischemia [255,256].

### 6.2. Motility

Excess zinc impairs mitochondrial trafficking in rat neurons [86,225]. Both zinc and pyrithione (a zinc ionophore) treatment and DTDP-induced cytosolic zinc release from MTs caused a significant decrease in mitochondrial movement, suggesting that the zinc sources responsible for these effects may be both extracellular and intracellular [86]. Moreover, zinc chelation with TPEN partially restored mitochondrial movement in cells treated with zinc and pyrithione, suggesting reversibility of this effect [86]. However, it is worth noting that the reversal of zinc inhibition of mitochondrial movement was only partially sensitive to zinc chelation. TPEN failed to restore mitochondrial movement when neurons were exposed to high concentrations of zinc or were exposed to the metal for longer durations. These data suggest the effects of zinc chelation on the restoration of mitochondrial movement are both concentration- and time-dependent, likely reflecting the activation of a downstream signaling cascade [86]. A later study, using primary cortical neurons, supported the idea that elevation of intracellular zinc disrupts mitochondrial transport in neurites [225]. Interestingly, this study showed that there may be spatial differences in the ability of zinc chelation to restore mitochondrial movement, as TPEN restored axonal but not dendritic mitochondrial movement [225]. However, in accordance with previous data, this study used a concentration of zinc that was previously shown to irreversibly impair mitochondrial movement in dendrites [86]. Thus, the extent of zinc inhibition of mitochondrial movement may be concentration-, time-, and location-dependent. Although the mechanism for zinc-induced decrease in mitochondrial movement is not yet fully elucidated, it may involve zinc activation of phosphatidylinositol 3-kinase (PI3K), as PI3K inhibition restored mitochondrial movement following zinc treatment at concentrations and durations insensitive to chelation [86]. It is worth noting that this effect was independent of changes in mitochondrial morphology or function [86]. 

## 7. Conclusions

**Figure 1:**
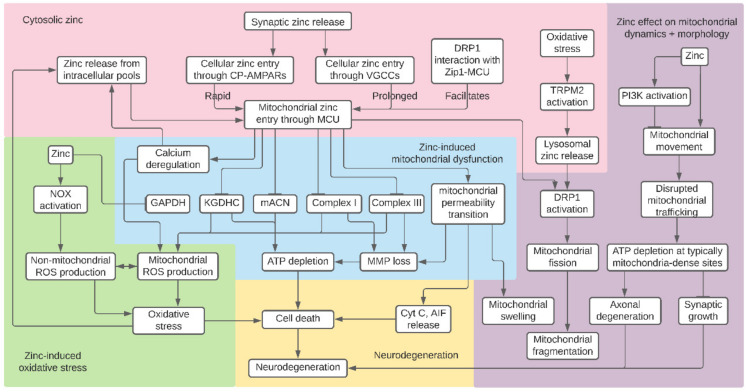
Summary of proposed mitochondrial effects of zinc. Synaptic zinc entry and release of zinc from cytosolic pools causes rises in intracellular zinc, which accumulates in the mitochondria. Zinc inhibition of components of glycolysis, the tricarboxylic acid (TCA) cycle, and the electron transport chain (ETC) result in mitochondrial membrane potential (MMP) loss and decreased ATP production. Other effects of excess zinc include reactive oxygen species (ROS) generation, mitochondrial permeability transition, and calcium deregulation. Finally, zinc inhibits mitochondrial movement and causes mitochondrial fragmentation by initiating excess fission. It is not clear if these pathways interact to induce their neurotoxic effects. Nevertheless, zinc-induced mitochondrial detriments may collectively result in cell death and neurodegeneration.

In this review, we have summarized existing knowledge on the myriad effects of zinc on mitochondrial function and dynamics in the context of neuronal health. Notably, zinc disrupts cell metabolism by inhibiting components of glycolysis, the TCA cycle, and the ETC. These zinc-induced impairments in bioenergetics lead to loss of MMP and decreased ATP production, which leads to neuronal cell death. Zinc also causes mitochondrial and non-mitochondrial ROS generation, which may work synergistically to induce oxidative stress. Zinc-induced mPTP opening is another facet of mitochondrial dysfunction, resulting in MMP loss, mitochondrial swelling, and release of apoptosis-promoting factors. It is important to note that many of these previously described mitochondrial defects follow mitochondrial zinc entry through the MCU and that zinc and calcium synergistically induce mitochondrial dysfunction. Finally, zinc impairs mitochondrial dynamics: zinc-induced fission results in mitochondrial fragmentation, and zinc inhibition of mitochondrial movement results in impaired mitochondrial trafficking. Although the potential interactions between these zinc-induced mitochondrial defects and their contributions to neurotoxic cascades are not fully understood, these effects may cause cell death and neurodegeneration. Zinc inhibition of cellular energy processes, subsequent mitochondrial defects, and changes in mitochondrial morphology and trafficking are summarized in Figure 1. 

While much progress has been made in understanding the effect of zinc on mitochondrial function, the work to elucidate the relationship between zinc, mitochondria, and neurodegeneration is far from over. As previously noted, mitochondrial defects can be cell type-dependent. For instance, brain mitochondria are less susceptible to mitochondrial permeability transition than liver mitochondria [114]. Thus, the findings from any non-neuronal studies described in this review should be interpreted cautiously when applied to neurons. Nevertheless, non-neuronal studies have historically provided insight into similar processes occurring in the brain.

Due to the lack of specificity of certain metal chelators and indicators in the past, there have been difficulties in assigning mitochondrial defects such as mitochondrial permeability transition to either zinc or calcium. In fact, the possible attribution of zinc-induced mitochondrial defects to calcium may underlie the clinical inefficacy of treating ischemia and other neurodegenerative diseases by targeting only calcium-associated excitotoxicity using NMDAR antagonists [257,258,259]. Thus, investigating zinc and calcium in relation to one another may provide new insights into the mechanisms by which these ions work together to contribute to mitochondrial defects such as mitochondrial permeability transition. Excitingly, the development of various novel tools and methods for detecting cytosolic and mitochondrial zinc [260], as well as more selective and fast chelators [20], may allow us to investigate the effects of zinc on mitochondria with greater specificity and accuracy than ever before. Prior use of FluoZin-3 in tandem with the calcium indicator fura-2FF has been particularly useful for determining the simultaneous effects of zinc and calcium [189,190,261]. Other recent detection methods include small molecule fluorophores, such as Zinpyr1 combined with genetically encoded mitochondrial localization sequences, to target fluorescent sensors to the organelle [262]. Fluorescence resonance energy transfer (FRET)-based biosensors, such as ZapCY1 and eCALWY4, which allow for ratiometric zinc measurement, have also been targeted to the mitochondria [263,264,265,266]. Lastly, in recent years, the development of the single fluorescent protein-based zinc sensors GZnP1 and GZnP2 has allowed researchers to visualize mitochondrial zinc dynamics on simpler imaging systems than those required for FRET sensors, and has even enabled estimations of labile zinc concentrations in specific mitochondrial regions such as the matrix and IMS [267,268]. This ability of GZnP2 to measure zinc concentrations across the IMM may be particularly useful in investigations of potential zinc transport across the IMM via ZnTs or ZIPs.

Finally, the contributions of zinc disruption of mitochondrial function, trafficking, and morphology to neurodegenerative disease pathologies are yet to be fully detailed. The role that zinc-induced mitochondrial dysfunction plays in ischemia has already been recognized [190]. However, zinc-induced mitochondrial defects likely play important roles in other neurodegenerative conditions, particularly those already linked to abnormal mitochondrial function and dynamics, such as PD, AD, HD, and ALS [131,235]. Indeed, although zinc-induced mitochondrial dysfunction has not been identified as a therapeutic target for these diseases, there have been new efforts to investigate how zinc relates to mitochondrial dysfunction in neurodegeneration [269]. Understanding the relationship between zinc and neuronal mitochondria provides insight into the myriad ways that zinc plays a role in mitochondrial and neuronal health under normal and pathological conditions. If zinc-induced mitochondrial deficits are found to play a role in neurodegenerative diseases, this may shed light on promising new targets for neuroprotection.

## Data Availability

Not applicable.
